# *LiST* modelling with monitoring data to estimate impact on child mortality of an ORS and zinc programme with public sector providers in Bihar, India

**DOI:** 10.1186/s12889-017-5008-y

**Published:** 2018-01-05

**Authors:** Jayachandran A. Ayyanat, Catherine Harbour, Sanjeev Kumar, Manjula Singh

**Affiliations:** 1 Avenir Health, New Delhi, India; 2 Children’s Investment Fund Foundation, London, UK; 3 Digital Green, New Delhi, India; 4 Children’s Investment Fund Foundation, New Delhi, India

**Keywords:** *LiST* modelling, ORS and zinc, Diarrhoea, Under-5 mortality, Bihar

## Abstract

**Background:**

Many interventions have attempted to increase vulnerable and remote populations’ access to ORS and zinc to reduce child mortality from diarrhoea. However, the impact of these interventions is difficult to measure. From 2010 to 15, Micronutrient Initiative (MI), worked with the public sector in Bihar, India to enable community health workers to treat and report uncomplicated child diarrhoea with ORS and zinc. We describe how we estimated programme’s impact on child mortality with Lives Saved Tool *(LiST)* modelling and data from MI’s management information system (MIS). This study demonstrates that using *LiST* modelling and MIS data are viable options for evaluating programmes to reduce child mortality.

**Methods:**

We used MI’s programme monitoring data to estimate coverage rates and *LiST* modelling software to estimate programme impact on child mortality. Four scenarios estimated the effects of different rates of programme scale-up and programme coverage on estimated child mortality by measuring children’s lives saved.

**Results:**

The programme saved an estimated 806–975 children under-5 who had diarrhoea during five-year project phase. Increasing ORS and zinc coverage rates to 19.8% & 18.3% respectively under public sector coverage with effective treatment would have increased the programme’s impact on child mortality and could have achieved the project goal of saving 4200 children’s lives during the five-year programme.

**Conclusions:**

Programme monitoring data can be used with *LiST* modelling software to estimate coverage rates and programme impact on child mortality. This modelling approach may cost less and yield estimates sooner than directly measuring programme impact with population-based surveys. However, users must be cautious about relying on modelled estimates of impact and ensure that the programme monitoring data used is complete and precise about the programme aspects that are modelled. Otherwise, *LiST* may mis-estimate impact on child mortality. Further, *LiST* software may require modifications to its built-in assumptions to capture programmatic inputs. *LiST* assumes that mortality rates and cause of death structure change only in response to changes in programme coverage. In Bihar, overall child mortality has decreased and diarrhoea seems to be less lethal than previously, but at present *LiST* does not adjust its estimates for these sorts of changes.

## Background

In 2013 alone more than 0.57 million children under five years of age died from diarrhoea [1]. Nearly one-fourth of these child deaths due to diarrhoea occurred in India [2]. As per international guidelines, in 2006, the India Academy of Paediatrics adopted the recommendations for treating diarrhoea with low osmolarity Oral Rehydration Salts (ORS) and zinc but implementation lagged behind [3]. In fact, diarrhoea still accounts for 10.5% of deaths among children under-5 in Bihar [4].

From 2010 to 2015, Micronutrient Initiative (MI), now Nutrition International conducted the Childhood Diarrhoea Management Programme (CDMP), also called the Diarrhoea Alleviation through Zinc and ORS Therapy (DAZT) project, in Bihar, Gujarat, and Uttar Pradesh. Children’s Investment Fund Foundation (CIFF) supported the Bihar programme [5]. The goal of the programme in Bihar was to reduce child morbidity and mortality related to diarrhoeal disease among children under-5 through improvements to public sector delivery of ORS and zinc for the treatment of childhood diarrhoea. MI implemented the programme in 15 demonstration districts and 23 scale-up districts of Bihar between August 2011 and August 2015. A team led by Johns Hopkins Bloomberg School of Public Health (JHSPH) and the Society for Applied Studies (SAS), New Delhi conducted a baseline survey in April – May 2011 and a follow-up survey in September – December 2013 [6]. JHSPH also used the *LiST* [7, 8] modelling software, to estimate how many children’s lives had been saved in the DAZT programme and its future potential for saving lives.

Over the years *LiST* has evolved to model more types of interventions and outcomes [9]. Studies have shown that the utility of *LiST* modelling to measure the potential effectiveness of an intervention or set of interventions related to mother and child health under scale up scenarios by estimating the number of lives saved [10–12]. It has been used to guide the strategic planning process in resource-poor settings with limited data inputs [13]. More recently, *LiST* has been used to identify high impact health interventions to end preventable deaths in mothers, newborns and stillbirths and to estimate their associated costs [14]. Furthermore, Walker and Walker [15] detail how *LiST* estimates deaths averted or lives saved by public sector interventions to reduce diarrhoeal deaths. Their findings demonstrate that *LiST* can be used as an alternative to large and expensive mortality impact studies.

Along with using *LiST* software, MI supported the state government to manage a robust management information system (MIS) to estimate the cases of diarrhoea and forecast the supply of ORS and zinc needed at different levels of service provision. Our study incorporates MIS data in *LiST* modelling to estimate the number of lives saved due to the ORS and zinc programme coverage. There are currently no studies of MIS data being utilized in *LiST* modelling, therefore our innovative approach has implications for scaling up cost-effective and impactful programmes.

This paper describes how we used the Lives Saved Tool (*LiST*) modelling software and MIS data to estimate the Bihar programme’s effect on child mortality. Through this study, we further highlight the possibilities and challenges of using *LiST* and MIS data to measure programmes.

## Methods

We used *LiST* version 5.31 to estimate the number of lives saved due to the specific levels of ORS and zinc coverage that the programme achieved, based on MI’s monitoring data. *LiST* requires three sets of inputs to project the impact of interventions on mortality: (1) measures of population-level health status including mortality and causes of death (available by default in *LiST*); (2) effect sizes [16, 17] of interventions and affected fractions of the population, and (3) intervention coverage (Table 1).

**Table 1:** Input data details for *LiST* modelling

*LiST* Input	Data Source	Date
Health status	Annual Health Survey (AHS) [18]	2010–11 (compiled for 15 programme intervention districts)
Effect sizes and affected fraction	International Journal of Epidemiology, 2010 and Research conducted worldwide provided by Child Health Epidemiology Reference Group (CHERG)	*LiST* version 5.31, updated on June 5, 2015
Intervention Coverage	Micronutrient Initiative (MI)‘s MIS and JHSPH survey data *(public sector only)*	MI’s MIS for 2013–14 and 2014–15; JHSPH, 2014

Rather than use primary data for both numerator (cases covered) and denominator (population affected) as the 2013 JHSPH *LiST* modelling did, our 2015 *LiST* modelling exercise used available data from MI’s monitoring data to estimate cases covered at the district level, and used other data sources to estimate the population affected.

### Input data

#### Population at risk

The target population of the intervention and the modelling is children aged 2–59 months from 15 demonstration districts.

#### Incidence of diarrhoea

The lower bound of diarrhoea incidence was estimated as 1.81 (diarrhoea incidence of 1.81 episodes/child/year) and a higher bound of 2.20 [19].

#### Public sector programme coverage

In our case the programme intervention for children with diarrhoea was those who received both ORS and zinc from a public-sector service provider. In the previous modelling exercise, JHSPH conducted two cross-sectional studies – baseline (2011) and a follow-up (2013) survey – and used coverage information from these two surveys to estimate the number of lives saved (or deaths averted) due to programme impact.

To estimate public sector coverage, we used three data sources i) MI’s MIS data, ii) JHSPH’s 2011 baseline and 2013 follow-up coverage data for ORS and zinc treatment from public health sector functionaries, and iii) the 2011 India Census, for estimates of population affected by childhood diarrhoea. We estimated the coverage rate numerator from MI’s MIS data and from JHSPH’s 2013 follow-up data, and the denominator from the 2011 India Census.

MI’s intervention was completed solely through public health functionaries in the intervention districts: Accredited Social Health Activists (ASHAs), *Anganwadi* workers (AWWs), Auxiliary Nurse Midwives (ANMs), Primary Health Centres and government hospitals. Front line health workers (ASHAs and AWWs) at the village level reported each diarrhoeal case and those treated with ORS and zinc within their respective geographies. Auxiliary Nurse Midwives reported cases and treatment at the sub-centre level. Monthly consolidated data was shared at a district and state level.

In 2011 and 2013, JHSPH and SAS surveyed caregivers of children who had had a diarrhoeal episode in the 14 days before the survey. The caregivers who reported having given their children ORS and/or zinc to treat the diarrhoea were asked about the source of the ORS and/or zinc. In addition to coverage estimates obtained from surveys, we also used coverage estimates inputs obtained from MIS. MIS coverage estimates are bound to overestimate since they are not based on cross-sectional data. To nullify such error, we used a multiplication factor (0.68) that was obtained by dividing coverage rates obtained from the JHSPH’s follow-up survey (2013) with the coverage estimate obtained from MIS in the same year. This adjustment was carried out for the coverage estimates calculated from MIS in subsequent years and inputted into the *LiST* modelling as listed in Table 2.

**Table 2:** Programme coverage estimates

	2010–11 (baseline)	2011–12	2012–13	2013–14	2014–15
ORS coverage (%) (based on MIS)			7.64	6.80	5.48
ORS coverage (%) (based on JHSPH survey)	1.83	2.95	5.2		
Multiplying factor			5.2/7.64 = 0.68		
Adjusted ORS coverage (%)	1.83	2.95	5.2	6.80×0.68 = 4.63	5.48×0.68 = 3.73

Similar adjustments were made while calculating zinc coverages.

The denominator of the coverage rate was estimated based on the number of children (2–59 months) who are affected with diarrhoea in the intervention areas. This was estimated based on the total number of children in that age group multiplied by the estimated incidence of diarrhoea in the age group. The total number of children aged 2–59 months was projected from a base population in 2011 using Census population [20] and adjusted with the annual exponential growth rate for these 15 demonstration districts. The current modelling used *LiST*’s default values for intervention efficacies and population affected fractions.

### Scenarios

**Fig. 1:**
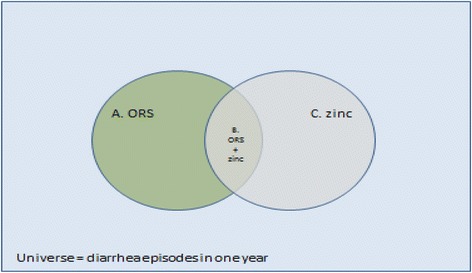
Graphical representation of diarrhoea prevalence and treatment

We developed four scenarios to establish appropriate mixes of coverage information. The following Table 3 and Fig. 1 provide the details of these scenarios.

**Table 3:** Details of scenarios considered for *LiST* modelling

Models	Descriptions
Scenario 1 *ORS and zinc (A intersection with C,* i.e.*, B))*	i. ORS coverage rate = Numerator is (Numbers treated with ORS only + Numbers treated with ORS and zinc) and ii. Zinc coverage rate = Numerator is (Numbers treated with ORS and zinc)
Scenario 2 ORS and zinc; ORS alone; zinc alone (A + C; A union with C, which includes B)	i. ORS coverage rate = Numerator is (Numbers treated with ORS only + Numbers treated with ORS and zinc) and ii. Zinc coverage rate = Numerator is (Numbers treated with ORS and zinc + those who received zinc alone)
Scenario 3 To achieve the 2010 CIFF estimate of 4200 additional number of deaths averted (cumulatively) *(B set to 4200)*	By working backwards, to achieve the 2010 CIFF estimate of 4200 additional cumulative number of deaths averted, what coverage rates for ORS and zinc would have been necessary after five years of programme intervention?
Scenario 4^a^ (Scenario 1 with 40% and 60% higher coverage rates)	This model will estimate the effect of larger coverage rates calculated from MIS data *(greatly improved coverage)* for ORS and zinc, with a “worst case” diarrhoea incidence scenario of 2.20 episodes/child/year.
Note	All scenarios have a denominator of total number of diarrhoea incidences/episodes in the population (among 2–59 month old children)

^a^This model doesn’t use JHSPH & SAS’s 2013 measured coverage rates but purely depends on MIS data, whereas, all other models inherently used JHSPH & SAS’s coverage rates

During the programme design phase, in 2010, CIFF estimated that the programme could save at least 4200 children’s lives. Scenario 3 of the 2015 modelling is a hypothetical as we estimated what programme coverage would have been necessary to reach this level of impact. This was worked out “backwards” by attempting several iterations in *LiST* to achieve the target of 4200 deaths averted cumulatively.

## Results

### Scenarios 1 and 2 – Actual programme coverage

**Fig. 2:**
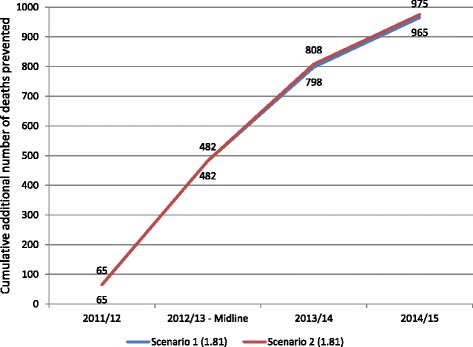
Cumulative number of additional deaths prevented in children under-five relative to baseline year (2010–11) due to ORS & Zinc program intervention – on the basis of incidence of 1.81 diarrhoeal episodes/child/year, Scenarios 1 & 2

Using the lower estimate of annual diarrhoeal incidence, *LiST* estimated that a total of between 965 (Scenario 1) and 975 (Scenario 2) additional lives were saved in children under 5, as a result of scaling up ORS and zinc treatment for diarrhoea through public sector providers (Fig. 2). The estimated additional number of lives saved in children under-5 age relative to impact year in 2010–11 are not significantly different.

**Fig. 3:**
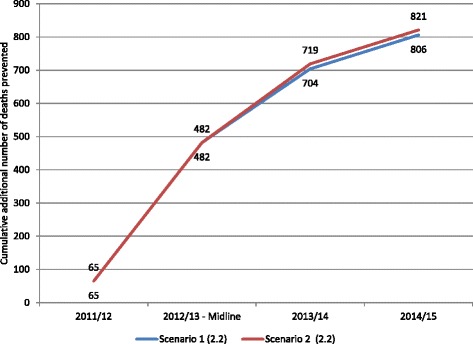
Cumulative number of additional deaths prevented in children under-five relative to impact year (2010–11) due to ORS & Zinc program intervention – on the basis of 2.20 diarrhoeal episodes/child/year, Scenarios 1 & 2

Using the higher estimate of annual diarrhoeal incidence, *LiST* estimated that a total of between 806 and 821 additional lives were saved among children under-5, as a result of scaling up ORS and zinc treatment for diarrhoea through public sector providers (Fig. 3). As with the lower estimate of annual diarrhoeal incidence, the *LiST* results for treatment scenarios 1 and 2 are not very different.

### Scenario 3 -- hypothetical model – Backwards coverage estimation to achieve design-stage impact target

To achieve a cumulative additional 4200 lives saved from expanding treatment of ORS and zinc in the public sector, our results *LIST* found that public sector coverage of ORS and zinc treatment would have had to expand from less than 2% in 2010–11 to nearly 20% in 2014–15 (Table 4).

**Table 4:** ORS and zinc coverage rates under public health sector to avert 4200 additional deaths in children under-5 years of age by intervention relative to baseline year – combined impact of ORS and zinc

	ORS Public sector Coverage (%)	Zinc Public sector Coverage (%)	No of additional deaths averted	Cumulative	Total No of children in 2–59 months^b^
2010–11	1.8	1.4	0	0	
2011–12	3.0	2.5	65^a^	65	
2012–13 – Midline	5.2	4.8	417	482	49,26,072
2013–14	14.6	13.5	1502	1984	49,90,391
2014–15	19.8	18.3	2222	4206	50,55,551

^a^Adjusted for the number of months/period programme implementation in different groups of districts

^b^Total number of children in 2–59 months estimated by MI for programme districts

### Scenario 4 – Hypothetical models -- 40% and 60% increased coverage (Fig. 4)

We constructed the final two scenarios to understand the worst possible diarrhoeal incidences (at 2.20 episodes/child/year) combined with the best possible programme coverage (i.e., 40% and 60% more public sector coverage than the coverage rates calculated from 2012 to 13 of MIS data in the year 2014–15, Table 5). This provides an opportunity to measure the impact of the programme intervention if the ORS and zinc coverage rates are peaked at these levels.

**Fig. 4:**
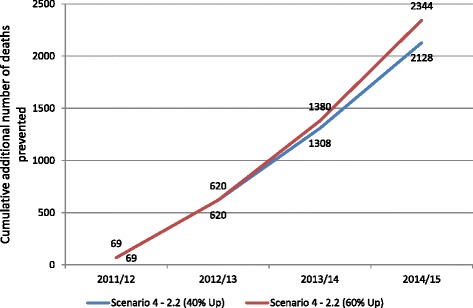
Cumulative number of additional deaths prevented in children under-five relative to impact year (2010–11) due to ORS & Zinc program intervention – on the basis of 2.20 diarrhoeal episodes/child/year with 40% and 60% increased ORS & Zinc coverage in MIS data, Scenario 4

**Table 5:** Public sector ORS and zinc coverage rates from MIS data; 40% and 60% greater than when measured 2013

	2012–13	40% increase by 2014–15	60% increase by 2014–15
ORS coverage	6.29%	8.80%	10.06%
Zinc coverage	5.88%	8.23%	9.40%

## Discussion

Public sector coverage of ORS and zinc would have had to have increased dramatically during the programme to avert more than 2000 deaths.

JHSPH’s 2013 projections based on *LiST* modelling found that at increased coverage levels– from 19.7% (from both the public and private sectors) in 2010 to 25.9% for ORS and from 3.7% in 2010 to 14.3% in 2015 for zinc – by 2015, more than 3400 diarrhoea related deaths among under-5 children in 15 demonstrated districts of Bihar would have been averted.

The 2015 *LiST* modelling results reported above, based on Scenarios 1–4, focus on public sector service coverage and are consistent with the 2013 JHU *LiST* modelling results. To have greater impact on child mortality, public sector coverage levels would have needed to increase quite ambitiously and remain increased as compared to the 2010–11 baseline levels. Had the programme been able to increase public sector coverage by 40%, more than 2100 children’s lives would have been saved, and had public sector coverage increased by 60%, more than 2300 lives would have been saved. To save 4200 children’s lives, the intervention would have had to increase its public sector ORS coverage from 1.8% in 2010–11 to 19.8% in 2014–15, and also its public sector zinc coverage from 1.4% in 2010–11 to 18.3% by 2014–15. These modelled coverage rates for ORS and zinc are ambitious given the field realities such as persistent stock outs of zinc and ORS in Bihar’s public sector made this sustained increase unfeasible [21].

## Limitations

Throughout our study, the *LiST* module was suitable for use at a sub-state level such as district in Bihar. However, the current *LiST* software present several limitations to our study as well.

Firstly, *LiST* does not provide any guidelines or modules on using MIS data to compute coverage rates for intervention programmes or extracting coverage rates separately from MIS data. Secondly, *LiST* does not include a joint coverage of both ORS and zinc though it is the recommended treatment for diarrhoea. Since *LiST* allows inputs for coverage rates of “ORS” (only) or “Zinc” (only) we had to accommodate the ORS + Zinc data into the *LiST* analysis by clubbing information pertaining to ORS + Zinc with ORS (only) and made two categories – ORS and zinc.

Thirdly, for the zinc (only) category, the field reports suggest that when health workers had no stock of ORS, they generally advised caregivers to give children oral rehydration therapies (ORTs) along with zinc, or get ORS from other sources. The potential impact of ORTs in combination with zinc is not captured in the model results, which may imply some level of additional effectiveness in treating diarrhoea cases.

Lastly, the overarching assumption in *LiST* is that any changes in the mortality rates and the cause of death structure is solely in response to changes in intervention coverages [22]. This assumption restricts the use of *LiST* model to short-term projection purposes not suitable for long-term projection as child mortality rates and cause of death distribution are rapidly changing.

## Conclusion

This article reports on the authors’ experience with using *LiST* software to model a programme’s impact on deaths from diarrhoea among children under-5 in Bihar. Our modelling assessed the impact of MI’s public sector ORS and zinc programme in 15 demonstration districts in Bihar using a population survey and programme MIS reporting data. We demonstrated that even with the limited data available in resource-poor settings, *LiST* software and utilization of MIS data can be a useful tool for programme evaluators and planners to estimate a programme’s impact on child mortality. For example, CIFF used *LiST* modelling to estimate the programme’s potential for impact on child mortality. The modelled impact results reported here (806 to 975) fell short of the 2010 CIFF projections which may be attributable to the challenges of the *LiST* software, programme execution, and data quality.

Our study also highlights the need to improve the *LiST* software. For example, *LiST* developers can provide appropriate guidelines on how to use MIS data to compute coverage rates for different intervention programmes and extract coverage rates from MIS data. The developers can also incorporate data collection for joint interventions such as treatment with both ORS and zinc. Finally, the software could expand the use of *LiST* model to long-term projection purposes as child mortality rates and cause of death distribution are rapidly changing.

Despite the improvements that need to be made to *LiST*, the software and utilization of MIS data should be seen as a complement rather than a replacement for household surveys. *LiST and MIS data* are viable options for estimating programme impact without the time and expense required to measure programme impact with a household coverage survey.

## Abbreviations

ANM: Auxiliary Nurse Midwife
ASHA: Accredited Social Health Activist
AWW: Anganwadi Worker
CIFF: Children’s Investment Fund Foundation
*LiST*: Lives Saved Tool
MI: Micronutrient Initiative
